# The effects of psychosocial stimulation on the development, growth, and treatment outcome of children with severe acute malnutrition age 6–59 months in southern Ethiopia: a parallel group cluster randomized control trial (EPSoSAMC study)

**DOI:** 10.1186/s12889-019-7916-5

**Published:** 2019-12-02

**Authors:** Tesfalem T. Tessema, Andamlak G. Alamdo, Tewodrose G. Yirtaw, Fana A. Deble, Eyoel B. Mekonen, Teklu G. Abessa, Tefera B. Lema

**Affiliations:** 1 Department of Public Health, St. Paul’s Hospital Millennium Medical College, Addis Ababa, Ethiopia; 2 College of Behavioral Sciences and Education, Jimma University, Jimma, Ethiopia; 3 College of Public Health and Medical Sciences, Jimma University, Jimma, Ethiopia

**Keywords:** Cluster randomized trial, Psychosocial stimulation, Under-five children, Severe acute malnutrition, Ethiopia

## Abstract

**Background:**

Severe Acute Malnutrition (SAM) remains a major cause of child mortality. To improve the management and survival of children the World Health Organization (WHO) endorsed the protocol for the management of SAM. The protocol suggested the integration of psychosocial stimulation as part of the medico-nutritional care process to prevent the long-term adverse developmental impact of the SAM. However, there is little scientific evidence behind the recommended stimulation intervention.

**Method:**

A parallel-group cluster-randomized controlled trial will be conducted among 144 children with SAM age 6–59 months in Southern Ethiopia. The study will have two groups where: children with SAM admitted in the intervention health facilities will receive psychosocial stimulation in addition to the routine inpatient care and for 6 months after discharge. Children with SAM admitted in the SC of the control health facilities will receive the routine inpatient care without psychosocial stimulation and home-based follow up for 6 months after discharge. All mothers/ caregivers will also receive uniform health education on child health-related issues. The primary outcome of the study will be child development while the secondary outcomes will include child growth and treatment outcome. All outcomes will be assessed four times: at enrollment, upon discharge from the SC, at 3 and 6 months of follow up. The data will be analyzed using STATA Version 15 Statistical Software. The anthropometric Z-scores and percentile of the median will be calculated child using WHO Anthro Version 3.2.2 Statistical Software. To assess the overall effect of the intervention by controlling other potential contributing factors, a generalized linear mixed model will be used.

**Discussion:**

The present study will have an important contribution in generating supplementary evidence regarding the effect of psychosocial stimulation interventions on the development and growth outcomes of children with SAM. The study will further address the impact of the intervention on treatment outcome indicators that are still under-researched areas requiring new scientific evidence.

**Trial registration:**

Pan African Clinical Trials Registry -PACTR201901730324304. Registered 25 November 2018, https://pactr.samrc.ac.za/TrialDisplay.aspx?TrialID=5739

## Background of the study

Globally approximately 151 million (22%) children under 5 years of age suffer from stunting and nearly 51 million (7.5%) were wasted in 2017. More than one-third of all stunted and more than one-quarter of all wasted children under 5 years of age are living in Africa [1]. According to the Ethiopian Demographic and Health Survey (EDHS) report, the prevalence of stunting, wasting and underweight among children under 5 years of age was 38, 10 and 24% respectively [2].

In view of the fact that malnutrition remains a direct or indirect cause of child mortality, the WHO developed the standard protocol to improve its management process and the survival of children. Considering the benefits of early child development programs in improving the long term developmental potential of children suffering from undernutrition, the protocols suggested the inclusion of emotional and physical stimulation through play programs during rehabilitation and after discharge [3]. However, few studies were conducted globally to examine the effect of the stimulation interventions provided in conjunction with the routine medico-nutritional care of children with SAM admitted in the nutrition rehabilitation unit. The study conducted in Bangladesh found out that the inclusion of the psychosocial stimulation interventions with the routine medico-nutritional care improved the mental development, motor development, weight-for-age Z-score and no change was noted for children behavior [4]. In Jamaica, the stimulation program showed a significant impact on the mental development of severely malnourished children [5]. In Ethiopia, the only study conducted among children with SAM admitted in the Stabilization Center (SC) of Jimma University Hospital revealed that the intervention significantly improved the gross motor functions during their hospital stay and fine motor functions with only small effect size after discharge from the hospital. However, it did not contribute significantly to linear growth and nutritional outcomes [6].

The reviewed studies have certain methodological issues that lead to very low-quality findings across important outcome indicators. A few of the limitations include the introduction of information contamination [6], the high level of lost to follow up (4,5&6), the lack of randomization [4].and unequal observation between the intervention groups [4]. Furthermore, in all of the reviewed studies, the full WHO recommendations on psychosocial stimulation [3, 7] were not implemented.

One of the goals of introducing stimulation and support intervention is to promote growth during the rehabilitation phase and to improve the treatment of severely malnourished children [8].

The reviewed studies did not adequately address the impact of the intervention on important treatment outcome indicators such as weight gain, duration of treatment, and the number of children discharged after recovery and the recurrence rate of malnutrition after discharge from the SC. Although positive findings have been observed from the few studies conducted in the area and the promotion of the stimulation intervention by organizations such as the WHO, the finding of a recent systematic review clearly indicated the need for further scientific evidence behind the inclusion of the stimulation intervention in the routine management of children with SAM [9].

## Hypothesis and objectives of the study

### Hypothesis

- It is hypothesized that children with SAM age 6–59 months admitted in the SC who receive psychosocial stimulation interventions with the routine inpatient care and for 6 months after discharge will have a significantly greater improvement on their development, growth and treatment outcomes compared to those who receive the routine care alone.

### Primary objective

To examine the effect of psychosocial stimulation interventions provided with the routine inpatient care and for 6 months after discharge on the development, growth and treatment outcome of children with SAM age 6–59 months in Southern Ethiopia.

### Secondary objectives

- To determine the effect of psychosocial stimulation interventions provided with the routine inpatient care and for 6 months after discharge on the development of children with SAM age 6–59 months
- To determine the effect of psychosocial stimulation interventions provided with the routine inpatient care and for 6 months after discharge on the growth of children with SAM age 6–59 months
- To determine the effect of psychosocial stimulation interventions provided with the routine inpatient care and for 6 months after discharge on the treatment outcome of children with SAM age 6–59 months

## Research methods and materials

### Study area and design

**Fig. 1:**
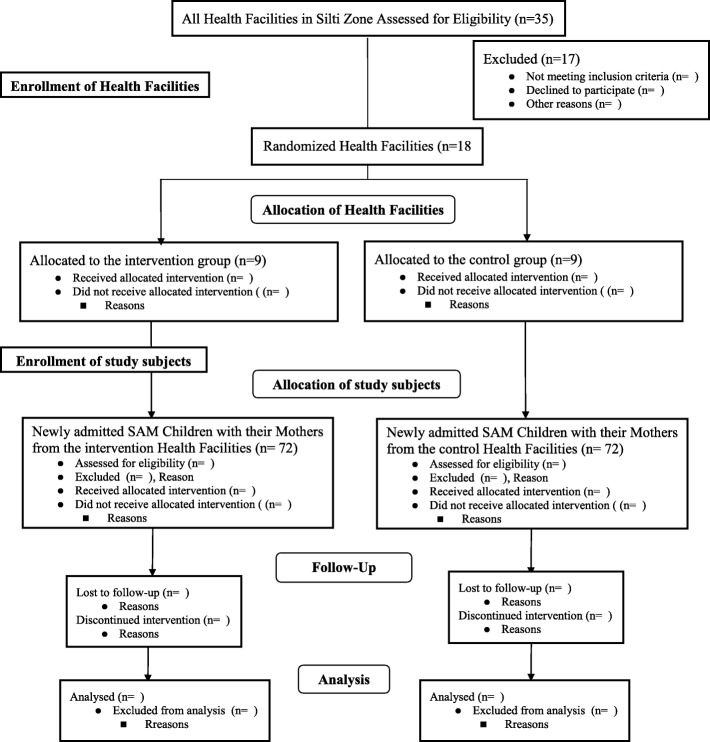
Flow Diagram for the Trial of Psychosocial Stimulation Intervention for children with SAM Age 6–59 Months, Southern Ethiopia, 2019

The study will be conducted in Silti Zone, Southern Regional State of Ethiopia. Administratively Silti Zone comprises nine District/Woredas with the one administrative town namely: Worabe that is located 175 km from Addis Ababa. In 2017, the total population of the Silti Zone was estimated to be 937,212 of which 482,527 were females and 454,685 were males. Nearly 837,207 (90%) of the total population lives in rural areas [10]. According to the report of the Zonal Health Office, there are thirty-five health facilities providing preventive and curative health services for their respective populations. Among these health facilities, nineteen of them have been providing inpatient care for children with SAM based on the national protocol for the inpatient management of SAM in their dedicated SC. The study will include 18 health facilities that have a dedicated SC. A Parallel-Group Cluster-Randomized Controlled Trial (RCT) will be used. The design is adopted because of its logistical feasibility for the study considering the difficulties to manage the use of different interventions for children admitted with similar cases in single SC. Furthermore, from our empirical observation mothers/ caregivers of children with SAM admitted in the SC usually create intimate living arrangements and relationships with each other possibly due to their extended stay in the SC of the health facility. They usually chat, prepare, and eat their food together. The intervention will further promote their intimacy since it has a daily informal group play session involving mothers/ caregivers and their children. If individual randomization is used within each health facility, the potential information contamination between the two groups could lead to the adoption of the psychosocial stimulation intervention by the control group, which will reduce the likelihood of finding substantial differences between the two groups of the study. The design is also appropriate considering the ethical issues that will be raised in relation to withholding intervention from some children and not others in the same SC of the health facility. The study protocol is prepared based on the SPIRIT guidelines and included all the recommended items of a clinical trial protocol (Fig. 1).

### Eligibility criteria for health facilities/clusters

All public health facilities in the study areas having a dedicated SC for the inpatient care of children with SAM and are willing to participate in the study will be eligible to take part in the study. At the time of submission, the Zonal Health office that has been managing all the health facilities agreed on the conduct of the study on behalf of the health facilities.

One health facility was excluded since it has recently established the SC and started providing inpatient care for children with SAM, unlike others.

### Eligibility criteria for children

During the recruitment period, all children with SAM age 6–59 months who will be showed up to the SC of selected health facilities for the inpatient care and fulfill the admission criteria of the Ethiopian guidelines for the management of acute malnutrition will be eligible study subjects. As per protocol, SAM in children age 6–59 months is diagnosed when; Weight–for-Length/Height (WFL/H) less than 70% of the WHO reference median or less than – 3 Z score and/or the presence of bilateral pitting edema and/or Mid Upper Arm Circumference (MUAC) < 11.5 cm. The inpatient admission criteria for children include having diagnosed with SAM and the presence of any of the medical complications listed in the national protocol or failed appetite tests or having diagnosed with SAM and referred from Outpatient Therapeutic Program (OTP) [11].

The inclusion criteria in the selection of eligible children with SAM;

- Children age 6–59 months with the diagnosis of SAM fulfilling the inpatient admission criteria based on the Ethiopian guidelines for the management of acute malnutrition
- The mother/ caregiver of the child has a plan to stay in the study area for at least 6 months after the discharge of her children from the SC
- The mother/ caregiver of the child agrees for the inpatient care of SAM during Phase 2 treatment in the SC
- The mother/ caregiver of the child agrees to present in the SC during the inpatient care

The exclusion criteria in the selection of eligible children with SAM;

- Children with the diagnosis of other chronic medical conditions such as tuberculosis and HIV/AIDS that could be identified by the health workers during admission or later
- Children with clinically apparent congenital abnormalities, obvious disabilities and sensory impairments (hearing and visual problems) that could affect outcome measurements and those children with twin birth
- Families of children living far from the health facilities included in the study

### Sample size determination and sampling technique

The sample size for the study is determined by considering the longitudinal (repeated measure from each subject over time) and clustered (study subjects nested in health facilities (clusters)) nature of the study. First, the sample size is determined by considering the longitudinal nature of the study using the formula for comparing two means [12] with the aim of detecting a 10% improvement from the mean + SD value of 15.8 + 4.4 for fine motor score of developmental domain which was obtained from similar study conducted in Ethiopia [6]. Four measurements are planned in the study. These include before the intervention when the child will be transferred from Phase I to the Transition Phase (baseline measurement), upon discharge from the SC of the health facilities and at three and 6 months. Thus, the number of time points of measurement taken from each study subjects; t = 4, type one error = 0.05, power = 90%, smallest meaningful difference d = 10% and the assumed correlation (r) of the repeated measures *r* = 0.02. Consequently, a sample of 44 children with the diagnosis of SAM satisfying the admission criteria to the SC per group will be required under individual randomization. The calculated sample size under individual randomization was further adjusted to account for the effect of clustering with an assumed Intracluster Correlation Coefficient (ICC) ρ = 0.05. Since this study is a trial with a fixed number of equal-sized clusters (k) the required sample size per group is obtained using the formula proposed by Hemming [13]. After considering, 20% of the calculated sample size for the potential dropout, 68 children will be required for each of the study groups. The corresponding number of individuals in each of the health facilities (clusters) is determined using the formula [13]. Accordingly, approximately eight study subjects from each of the study health facilities (clusters) will be required making the final sample 72 children with SAM for each of the study groups. Thus, 144 children with SAM age 6–59 months satisfying the inpatient admission criteria will be recruited and included in the two study groups. Based on the recommendation of Hemming [13] this trial is feasible since the number of clusters k = 9 per group is greater than the product of the number of individuals required under individual randomization (*n* = 44) and ICC (ρ = 0.05). Based on the report of the Silti Zone Health Office, the achievement of the target sample of children with SAM is feasible during the recruitment period.

### Randomization

There are a total of, *K* **=** 18 study health facilities (clusters), k = 9 clusters will be used in each of the two intervention groups. The interventions will be allocated randomly to the study health facilities (clusters) by using a table of random numbers by an independent randomization manager who will not know anything about neither the interventions nor the health facilities (clusters). The allocation code will be concealed from the randomization manager to either of study groups using sealed opaque envelopes.

### Personnel for the study

Health workers who will be recruited from the SC of each of the health facilities will take the responsibility for the recruitment; follow up of children during the SC admission period, for supporting the intervention workers/play guides and for the collection of enrollment and other data. Depending on the caseload in the SC of the health facility, one or two health workers will be included in the study team from each of the health facilities.

For each of the health facilities, one young female intervention worker/ play guide who have completed at least grade 10 will be recruited from the local community considering their child-friendliness. In the intervention health facilities, the intervention workers/ play guides will assume the responsibility of facilitating the stimulation intervention during the inpatient care and for the period of 6 months after discharge through a home visit. They will also provide health education, conduct home-based follow up, collect follow up data, participate in the preparation of suitable toys from locally available materials, help mothers/ caregivers and other family members, and equip them with the essential skills for the implementation of the intervention. For each of the control health facilities, the intervention worker will only be responsible for providing health education, conducting home-based follow-up, and collection follow-up data. A separate mobile team of testers/ outcome assessors will be recruited considering their academic background and previous experience to collect anthropometric data and administer developmental tests. They will be unaware (Blind) to the allocation status of the children and the health facilities to avoid measurement bias.

Experienced supervisors will be recruited to oversee the activities of the health workers, the team of testers/ outcome assessors and the intervention workers. The field-level project coordinator under the direct guidance of the principal investigator will be responsible for the overall coordination and follow up.

### Training of the study team

Intensive training will be given for the study personnel by a team of senior trainers. Separate training will be provided for each of the study teams considering the allocation status of their health facilities, their role in the study and their professional experience. For the intervention health facilities, health workers will be trained on the basis of psychosocial stimulation and the study procedures including recruitment and data collection procedures, facilitation of psychosocial stimulation, and facility-level follow up procedures. The training of the intervention workers/play guides will focus on the basics of childcare, child growth, and development, about the communication methods and other intervention-related issues such as the study procedures, the facilitation of the stimulation intervention and follow up procedures. For the control health facilities, the training of both the health workers and intervention workers/ play guides will not include the stimulation intervention related topics. The measurement team will also be trained on the standards of anthropometric measurement and administration of development tests. In all the training sessions, the trainees will go out to the field to practice the practical components of the training.

### Recruitment procedure

Prior to the recruitment, a dedicated play corner will be established at each of the intervention health facilities and equipped with all the essential materials including age and developmentally appropriate play materials that will be used for providing stimulation intervention through play program. Intensive efforts will be undertaken to make the atmosphere in the SC room and the play corner relaxed, cheerful and welcoming.

In the 2 months recruitment period, health workers will screen children with SAM who will be shown up to the SC of the selected health facilities and recruit the first eight children with their mothers/ caregivers based on the study inclusion criteria. The health workers will discuss with the caretakers of all children fulfilling the inclusion criteria about the basic concepts of child nutrition, child growth and development and the details of the study including the involvement of the mothers/caregivers, the associated training need, about the required measurements and follow up procedures. Then, they will invite mothers/ caregivers to participate in the study with their child. After having received written informed consent from the mothers/ caregivers for their voluntary participation with their child, they will collect enrollment data.

### The intervention and follow-up

The intervention and follow up of the study subjects will take place for 8 months. In the intervention health facilities: children with SAM age 6–59 months will receive psychosocial stimulation in addition to the routine inpatient care in the SC and for 6 months after discharge through home-based follow up. In this study, the full WHO recommendations on emotional and physical stimulation [3, 7] will be implemented in the intervention health facilities. After the initial phase of treatment (Phase-I), children with SAM will participate in the psychosocial stimulation intervention at the regular schedule during the transition and phase 2 of the inpatient care and for 6 months after discharge through home-based follow up. In the SC, the child will spend prolonged periods on large play mats with other children, mothers and/or an intervention worker/ play guide on a daily basis to facilitate informal group play sessions. Each of the mother/ caregiver and their child will also participate in a half-hour of individual play sessions on a daily basis with an intervention worker/ play guide worker. Since, an average of 2–3 weeks are required to offer the full inpatient care (Phase 1, Transition Phase and Phase 2) in the SC, each child will receive about 12–19 individualized stimulation sessions in the SC after Phase 1 medico nutritional care. The play activities and toys will be selected to develop both motor and language skills considering the age and the level of child development. Accordingly, each play session will include language activities, motor activities, and activities with toys.

Toys will be always available in the child’s cot and room, as well as in the play area and they will be changed frequently. Inexpensive, safe and washable toys made from locally available materials such as cardboard boxes, plastic bottles, and tin cans and Western toys will be used. For those children who are unable to move, passive limb movements and splashing in a warm bath will be used to promote mobility. For other children, play activates will include rolling on a mattress, running after and tossing a ball, climbing stairs and walking. The duration and intensity of physical activities will be increased as the child’s nutritional status and general condition improve. A guiding curriculum with detailed play activities arranged by the age and the level of child development will be used to assist intervention workers/ play guides in the delivery of the intervention. The curriculum is prepared based on the Ethiopian protocol for the management of SAM [14] and recommendations of the WHO [3, 7]. The mothers/ caregivers will also be encouraged to play and chat with their children, asked for any questions they may have and given new skills and positive feedbacks to continue the activities between the scheduled stimulation sessions. They will be given complete information about the importance of adhering to the study procedures at discharge from the SC of the health facilities.

In the control health facilities, SAM children age 6–59 months will receive the routine inpatient care without psychosocial stimulation and home-based follow up for 6 months after discharge through home-based follow up.

In the 6 months follow up period after the discharge from the inpatient care, all children will be visited at home for five times at the end of 1st week, 2nd weeks, 1st month, 3rd months, and 6th months based on the recommended follow-up schedule of the WHO [3]. For the intervention groups, the intervention workers/ play guides will facilitate a half-hour individual stimulation session in each of the scheduled home visits where they will play with the child, the mother/ caregiver, and other family members if any. They will remind mothers/ caregivers on the importance of following study procedures and left extra play materials at home to enhance the interest of the child to the play activities.

The health education provided in Ethiopian public health facilities luck uniformity due to factors such as the absence of standardized training curriculum, health education manual and the variation in the training level of health providers. Therefore, mothers/ caregivers under both intervention and control group will also receive health education on the childcare such as child nutrition, child feeding, sanitation, and child growth and development during the inpatient care and for the period of 6 months after discharge. The health education manual consisting of key messages and the delivery schedule will be used to guide intervention workers in the delivery of uniform educational activities across all health facilities. The manual is prepared based on the available documents used in the Ethiopian context [11, 14–16].

### Outcome measurement and tools

All outcomes will be assessed four times throughout the follow-up period. These include before the intervention when the child will be transferred from Phase I to the Transition Phase (baseline measurement), upon discharge from the SC of the health facilities and at 3 and 6 months where all mothers/ caregivers will be asked to bring their child to the health facilities for the latter two measurements. As per recommendations of the pilot study conducted to assess the feasibility and reliability of the sequence of various measurements, measurements requiring greater concentration (developmental tests) will be administered first that will be followed by the anthropometric measurements which will require less concentration [17].

### Primary outcome

The primary outcome of the study will be child development. The five areas of child development including personal-social, fine motor, language, gross motor, and social-emotional skills will be measured. The first four of the developmental dimensions will be measured using the Denver II-Jimma which is a culturally adapted, standardized and translated tool [18]. The tool was previously used in a number of studies to assess the developmental performance of Ethiopian children [19–21]. For each child, the performance score that refers to the sum of all items for each developmental domain will be calculated. The remaining social-emotional dimensions of the child development will be measured using parent completed Ages and Stages Questionnaire: Social-Emotional (ASQ: SE). The tool was adapted and translated for the Ethiopian context and used previously [19–21]. The child’s total score will be calculated by adding up the points of all items on the questionnaire.

### Secondary outcome

The secondary outcomes of the study will include growth and the treatment outcomes of children with SAM. In order to assess the growth performance of the children, weight, height/length and MUAC will be measured following the standard procedure based on the Ethiopian national protocol for the management of SAM [14].

To determine treatment outcome, the death rate, recovery/cure rate, defaulter rate, non-responder rate, the average rate of weight gain, and the mean length of stay of children in the SC will be calculated based on the Ethiopian guidelines for the management of acute malnutrition [11]. The proportion of cases that will have relapsed SAM within the 6 months follow up period will be calculated. Children will be considered to have a relapse, when their WFL/H is less than 70% of the WHO reference median or less than – 3 Z score and/or having bilateral pitting edema and/or MUAC < 11.5 cm.

Detail data on the socio-demographic situation of the family and other relevant data such as the mothers/ caregivers’ knowledge of childcare, dietary data, household food insecurity data, and data on mother/ caregiver-child relationship, the presence of family conflict, and the previous medical conditions will be collected using a structured questionnaire. The health workers will administer the Amharic version of the questionnaire to the mothers/ caregivers through a face-to-face interview during the transition phase of the inpatient management of SAM when the clinical condition of the child is improved and the mothers/ caregivers become more relaxed. To collect follow up data, a separate structured questionnaire will be used during home visits. Data on the adherence of mothers/ caregivers under the intervention group towards the implementation of the simulation activities, the reason for non-adherence if any, the support and involvement of other family members in the stimulation interventions will also be gathered in the follow-up visits (Additional file 1 & Additional file 2).

### Data management and analysis

Two independent data entry operators will check, code, and enter the collected data into Epi Info Version 7 Statistical Software. STATA Version 15 Statistical Software will be used for the statistical analyses. The anthropometric Z-scores and percentile of the median will be calculated using the WHO Anthro Version 3.2.2 Statistical Software. Proportions, percentages, measures of central tendency together with measures of dispersion will be used to summarize the collected data. For each test item in the developmental measures, a binary outcome variable with the categorical score of a pass or fail will be created where; pass will be considered for items that the child will be tested and passed while fail will be considered for items that the child will be tested and failed or refused. No opportunity to perform will be considered for items that the child will not be tested for due to various reasons including medical conditions, which will be treated as missing values. A numerical score will be computed by adding up the points of all items for each of the developmental dimensions. For the social-emotional dimensions of the child development that will be measured using ASQ: SE, a numerical score will be computed by adding up the points of all items on the questionnaire. From the anthropometric data of weight, height, and MUAC, the nutritional status of children will be determined. The proportion of cases in either group of the study that will be recovered, defaulted, and dead or relapsed within the study period will be also calculated and reported.

In a cluster RCT, participants within the same cluster may be more similar than those in different clusters, which may lead to the correlation of observations within the clusters. Failure to account this in the analysis and the subsequent use of standard statistical methods will make the resulting standard error of the intervention effect too small, along with the confidence interval and *P*-value [22]. In all the analyses that will be conducted using the data of all randomized study subjects, the clustering nature of the data will be considered. In order to assess the overall effect of the intervention by controlling other potential contributing factors, a Generalized Linear Mixed Model (GLMM) that is flexible to address the outcome variables to be measured in this study will be used.

The model is appropriate for the present study considering the correlated nature of the repeated measurements from each study subject and from the hierarchical structure of data that study subjects nested in health facilities (clusters). In all the analysis variables with a *p*-value < 0.05 will be considered statistically significant.

### Plan for dissemination of the findings

The finding of the study will be reported according to the CONSORT statement for reporting a cluster-randomized trial [23]. Right after the last outcome measurement, written feedback will be given to the mothers/ caregivers with a verbal explanation about the overall findings of the developmental tests and anthropometric measurement along with a brochure explaining child health, growth, development and related issues. The finding of the study will be shared with all concerned bodies including the Ethiopian Federal Ministry of Health, local health offices, and other partners. We will also present the finding of the study on relevant conferences and publish on open access peer-reviewed journals for wider dissemination to the scientific community. In all manuscripts submitted for publication, authorship will be granted to individuals who will have substantive contributions to the design, conduct, interpretation, and reporting of the study based on the authorship criteria of the International Committee of Medical Journal Editors.

### Trial steering committee

The Trial Steering Committee (TSC) will monitor the overall process of the research activities ensuring that the research is conducted according to the protocol. The committee consisting of an independent chair and other independent members will meet regularly to discuss the performance of research activities and review the progress. The committee will also be responsible to recommend the consideration of appropriate measures in the conduct of the study such as amendments to the study protocol or stopping or extending the study based on relevant evidence.

## Discussion

Globally, few studies were conducted to examine the effect of psychosocial stimulation interventions provided in conjunction with the routine nutritional care of children with SAM.

The present study will have an important contribution in generating supplementary evidence regarding the effect of psychosocial stimulation interventions on the development and growth outcomes of children in resources poor public health facilities. The study will further address the impact of the intervention on treatment outcome indicators that are still under-researched areas requiring new scientific evidence. Unlike most of the previous studies conducted in the area, the full WHO recommendations on psychosocial stimulation will be implemented in the present study.

### Trial status and the time schedule of execution

Currently, we have made a discussion with the Silti Zone Health office, which has been managing the eighteen health facilities, and the office expressed an interest to participate in the study. At the time of submission, the recruitment of study subjects is not yet started. The whole study will take an estimated 14 months. The preparatory works such as the selection of the research team, their training, and logistic arrangement will take approximately 3 months. The recruitment, the intervention and follow up of the study subjects will take place for 8 months duration beginning from November 2019. The activities of the final phase of the study such as data analysis, report write up and manuscript preparation will take an estimated 3 months.

## Supplementary information

**Additional file 1.** Data Collection Questionnaire

**Additional file 2.** Follow Up Data Collection Questionnaire

## Data Availability

The data set of the current study will be available from the corresponding author on a reasonable request.

## Abbreviations

ASQ:SE: Ages and Stages Questionnaire: Social-Emotional
EDHS: Ethiopian Demographic and Health Survey
GLMM: Generalized Linear Mixed Model
ICC: Intracluster Correlation Coefficient
IRB: Institutional Review Board
MUAC: Mid Upper Arm Circumference
OTP: Outpatient Therapeutic Program
RCT: Randomized Controlled Trial
SAM: Severe Acute Malnutrition
SC: Stabilization Center
TSC: Trial Steering Committee
WFL/H: Weight–for-Length/Height
WHO: World Health Organization

## References

[1] UNICEF, WHO, World Bank Group (2018). Levels and Trends in Child malnutrition: Key findings of the 2018. Edition of the Joint Child Malnutrition Estimates.

[2] Central Statistical Agency [Ethiopia] and I. Ethiopia Demographic and Health Survey 2016. Addis Ababa: CSA and ICF. p. 2016.

[3] WHO (1999). Management Of Severe Malnutrition : A Manual for Physicians and Other Senior Health Workers.

[4] Nahar B, Hamadani JD, Ahmed T, Tofail F, Rahman A, Huda SN (2009). Effects of psychosocial stimulation on growth and development of severely malnourished children in a nutrition unit in Bangladesh. Eur J Clin Nutr [Internet].

[5] Grantham-McGregor S, Schofield W, Powell C (1987). Development of severely malnourished children who received psychosocial stimulation : six-year follow-up. Pediatrics.

[6] Abessa TG, Worku BN, Wondafrash M, Girma T, Valy J, Lemmens J (2019). Effect of play-based family-centered psychomotor / psychosocial stimulation on the development of severely acutely malnourished children under six in a low- income setting : a randomized controlled trial. BMC Pediatr.

[7] Ashworth A, Schofield EC, Khanum S, Jackson A (2003). Guidelines for the inpatient treatment of severely malnourished children.

[8] Kerac M, Mcgrath M, Grijalva-Eternod C, Bizouerne C, Saxton J, Bailey H (2010). Management of Acute Malnutrition in infants (MAMI) project: technical review.

[9] Daniel AI, Bandsma RH, Lytvyn L, Voskuijl WP, Potani I, van den HM. Psychosocial stimulation interventions for children with severe acute malnutrition: a systematic review. J Glob Health. 2017;7(1):1–12. 10.7189/jogh.07.010405 PMC5441448 28567278

[10] FDRE-CSA. Population Projection of Ethiopia for All Regions At Wereda Level from 2014–2017: Addis Ababa; 2013.

[11] Ethiopian FMoH. Guidelines for the Management of Acute Malnutrition: Addis Ababa; 2016.

[12] Diggle PJ, Heagerty P, Liang K-Y, Zeger SL (1994). Analysis of longitudinal data [internet].

[13] Hemming K, Girling AJ, Sitch AJ, Marsh J, Lilford RJ (2011). Sample size calculations for cluster randomised controlled trials with a fixed number of clusters. BMC Med Res Methodol [Internet].

[14] Ethiopian FMoH. Protocol for the Management of Severe Acute Malnutrition: Addis Ababa; 2007.

[15] Ethiopian FMoH. Job aid for maternal nutrition and age Appropriate Infant & Young Child Nutrition: Addis Ababa; 2016.

[16] Ethiopian FMoH. National Guideline on adolescent, maternal infant and young child nutrition: Addis Ababa; 2016.

[17] Yousafzai AK, Obradović J, Rasheed MA, Rizvi A, Portilla XA, Tirado-Strayer N (2016). Effects of responsive stimulation and nutrition interventions on children’s development and growth at age 4 years in a disadvantaged population in Pakistan: a longitudinal follow-up of a cluster-randomised factorial effectiveness trial. Lancet Glob Heal.

[18] Abessa TG, Worku BN, Kibebew MW, Valy J, Lemmens J, Thijs H (2016). Adaptation and standardization of a Western tool for assessing child development in non-Western low-income context. BMC Public Health [Internet].

[19] Worku BN, Abessa TG, Wondafrash M, Lemmens J, Valy J, Bruckers L (2018). Effects of home-based play-assisted stimulation on developmental performances of children living in extreme poverty: a randomized single-blind controlled trial. BMC Pediatr.

[20] Abessa TG, Bruckers L, Kolsteren P, Granitzer M (2017). Developmental performance of hospitalized severely acutely malnourished under-six children in low- income setting. BMC Pediatr.

[21] Worku BN, Abessa TG, Wondafrash M, Vanvuchelen M, Bruckers L, Kolsteren P (2018). The relationship of undernutrition/psychosocial factors and developmental outcomes of children in extreme poverty in Ethiopia. BMC Pediatr.

[22] McKenzie J, Ryan RDTG (2014). Cochrane Consumers and Communication Review Group : Cluster randomised controlled trials.

[23] Campbell Marion K, Elbourne Diana R, Altman Douglas G (2004). CONSORT statement: extension to cluster randomised trials. BMJ.

