# Supplementary material for: The effects of psychosocial stimulation on the development, growth, and treatment outcome of children with severe acute malnutrition age 6–59 months in southern Ethiopia: a parallel group cluster randomized control trial (EPSoSAMC study)
Source: BMC Public Health. 2019 Dec 2;19:1610. doi: 10.1186/s12889-019-7916-5 (PMC6889618; doi:10.1186/s12889-019-7916-5)
Supplement: Supplementary file 1 — Additional file 1. Data Collection Questionnaire [file 12889_2019_7916_MOESM1_ESM.docx]

Additional File 1: Data Collection Questionnaire

| **Section I ; General Information** | | | | |
| --- | --- | --- | --- | --- |
|  | Child's unique ID Number | |  | |
|  | Child's Name | |  | |
|  | Name of Data Collector | |  | |
|  | Name of Supervisor | |  | |
|  | Date of data collection | | (DD/M/Y-GC) | |
|  | Health Facility Name | |  | |
| **Section II ; Demographic And Socio-Economic Information** | | | | |
| **Code** | **Questions and Filters** | **Coding Categories** | | **Skip** |
|  | Primary Caretaker | 1. Mother 2. Father 3. Relatives 4. Others (Specify) | |  |
|  | Age of the Mother | (In Complete Years) | |  |
|  | Religion | 1. Orthodox 2. Catholic 3. Protestant 4. Muslim 5. Others (Specify) | |  |
|  | Marital status | 1. Married 2. Living together 3. Divorced/Separated 4. Widowed 5. Single | |  |
|  | Ever attended school (for mothers) | 1. Yes 2. No | |  |
|  | Educational status of the mothers | 1. Primary education 2. Secondary education 3. Technical/Vocational 4. Higher education | |  |
|  | Current occupation of the mother | 1. Farmer 2. Self-employed (Non-farming) 3. House wife 4. Government employed 5. Private employed 6. NGO employed 7. Daily laborer 8. Student 9. Others (Specify) | |  |
|  | Ever attended school (for fathers) | 1. Yes 2. No | |  |
|  | Educational status of the fathers | 1. Primary education 2. Secondary education 3. Technical/Vocational 4. Higher education | |  |

|  | | Current occupation of the fathers | | 1. Farmer 2. Self-employed (non-farming) 3. Government employed 4. Private employed 5. NGO employed 6. Daily laborer 7. Student 8. Others (Specify) | | | | | | | | | | | | | | |  | |
| --- | --- | --- | --- | --- | --- | --- | --- | --- | --- | --- | --- | --- | --- | --- | --- | --- | --- | --- | --- | --- |
|  | | Head of the household | | 1. Mother 2. Father 3. Others (Specify) | | | | | | | | | | | | | | |  | |
|  | | Number of persons living under the same roof with the child | |  | | | | | | | | | | | | | | |  | |
|  | | Number of children in the household | |  | | | | | | | | | | | | | | |  | |
|  | | Number of children age below 5 years in the household | |  | | | | | | | | | | | | | | |  | |
|  | | The birth order of [the name of the child]? | |  | | | | | | | | | | | | | | |  | |
|  | | How many months of difference between [the name of the child] and the older sibling? | |  | | | | | | | | | | | | | | |  | |
|  | | Number of children the child meets in neighbor | |  | | | | | | | | | | | | | | |  | |
|  | | Frequency of the child’s interaction with other children | | 1. Always 2. Sometimes 3. Never interacts with others | | | | | | | | | | | | | | |  | |
|  | | Mother/Primary caretaker –child interaction | | 1. Yes 2. No | | | | | | | | | | | | | | |  | |
|  | | Mother -child interaction time | | 1. During feeding and toileting 2. Out of working hours 3. Always together | | | | | | | | | | | | | | |  | |
|  | | Other family members-child interaction | | 1. Yes 2. No | | | | | | | | | | | | | | |  | |
|  | | Other family members-child interaction time | | 1. During feeding and toileting 2. Out of working hours 3. Always together | | | | | | | | | | | | | | |  | |
|  | | A person more often attached with the child | | 1. Elder brother/sister 2. Younger brother/sister 3. A child less than 11 years of age 4. Grandparents 5. The child is lonely | | | | | | | | | | | | | | |  | |
|  | | Time spent on play by the child | | 1. Always 2. Sometimes 3. Very rarely 4. Does not play at all | | | | | | | | | | | | | | |  | |
|  | | Facilities for play | | 1. Has different play material 2. Has play corner arranged for him 3. Plays cultural dances, songs and riddles with family | | | | | | | | | | | | | | |  | |
|  | | Child feeding condition | | 1. Gets age appropriate diets 2. Gets what is available for family 3. Has very poor appetite for food 4. does not get sufficient food | | | | | | | | | | | | | | |  | |
| **Section III ; Housing Condition and Household Assets** | | | | | | | | | | | | | | | | | | | | |
|  | | What is the main material of the walls? | | 1. Natural materials (cane, wood, mud) 2. Plank 3. Stone with mud 4. Stone with cement/bricks 5. Others (Specify) | | | | | | | | | | | | | | |  | |
|  | | What is the main floor material? | | 1. Natural floor (earth/sand/dung) 2. Rudimentary floor (wood/palm/bamboo 3. Finished floor (Polished wood, vinyl, tiles, cement, carpet) 4. Others (Specify) | | | | | | | | | | | | | | |  | |
|  | | What is the main material of the roof: | | 1. Iron sheets or tiles; 2. Thatch/grass or leaves; 3. Others (Specify) | | | | | | | | | | | | | | |  | |
|  | | How many rooms do you have in your household? | | Number | | | | | | | | | | | | | | |  | |
|  | | Does your house have windows? | | 1. No  2. Yes | | | | | | | | | | | | | | |  | |
|  | | Is the house connected to electricity | | 1. No  2. Yes | | | | | | | | | | | | | | |  | |
|  | | Do you own land? | | 1. Yes 2. No | | | | | | | | | | | | | | |  | |
|  | | How many hectares of land do you own? | | (ha) | | | | | | | | | | | | | | |  | |
|  | | In this household are there the following household materials and Livestock (Wealth index measuring tools): | | | | | | | | | | | | | | | | | | |
|  | | **Household Materials** | **1-Yes** | | **2-No** | | | |  | **Livestock** | | | | | **1-Yes** | | | | | **2-No** |
|  | | - 1. Bed |  | |  | | | |  | - 1. Ox | | | | |  | | | | |  |
|  | | - 1. Mattress |  | |  | | | |  | - 1. Cow | | | | |  | | | | |  |
|  | | - 1. Table |  | |  | | | |  | - 1. Young bull | | | | |  | | | | |  |
|  | | - 1. Chair |  | |  | | | |  | - 1. Heifer | | | | |  | | | | |  |
|  | | - 1. Refrigerator |  | |  | | | |  | - 1. Sheep | | | | |  | | | | |  |
|  | | - 1. Cell phone (Mobile) |  | |  | | | |  | - 1. Goat | | | | |  | | | | |  |
|  | | - 1. Television |  | |  | | | |  | - 1. Donkey | | | | |  | | | | |  |
|  | | - 1. Radio |  | |  | | | |  | - 1. Horse | | | | |  | | | | |  |
|  | | - 1. Electrical stove |  | |  | | | |  | - 1. Mule | | | | |  | | | | |  |
|  | | - 1. Car |  | |  | | | |  | - 1. Hen | | | | |  | | | | |  |
|  | | - 1. Bicycle |  | |  | | | |  |  | | | | |  | | | | |  |
|  | | - 1. Motor |  | |  | | | |  |  | | | | |  | | | | |  |
|  | | - 1. Cart |  | |  | | | |  |  | | | | |  | | | | |  |
|  | | - 1. Beehives |  | |  | | | |  |  | | | | |  | | | | |  |
|  | | Taking the past six months, tell me what the average earning (birr) of the household have been? | | | Per week ______________________ (birr)  Per month _____________________ (birr) | | | | | | | | | | | | | | | |
| **Section IV ; Obstetric History** | | | | | | | | | | | | | | | | | | | | |
|  | | Are you pregnant now? | | | | | 1. Yes 2. No | | | | | | | | | |  | | | |
|  | | Are you currently attending antenatal care for your current pregnancy? | | | | | 1. Yes 2. No | | | | | | | | | |  | | | |
|  | | Did you attend antenatal care for your last pregnancy? | | | | | 1. Yes 2. No | | | | | | | | | |  | | | |
|  | | How many times did you attend antenatal care for your last pregnancy? | | | | | (Number of times) | | | | | | | | | |  | | | |
|  | | For your last pregnancy, were you counseled about infant feeding methods? | | | | | 1. Yes 2. No | | | | | | | | | |  | | | |
|  | | Which feeding methods were you counseled about? | | | | | 1-Yes | | | | | | 2-No | | | | | | | |
|  | | - 1. Breastfeeding | | | | |  | | | | | |  | | | | | | | |
|  |  | - 1. Formula feeding | | | | |  | | | | | |  | | | | | | | |
|  |  | - 1. Complementary feeding | | | | |  | | | | | |  | | | | | | | |
|  |  | - 1. Bottle feeding | | | | |  | | | | | |  | | | | | | | |
|  |  | - 1. Cow milk feeding | | | | |  | | | | | |  | | | | | | | |
|  |  | - 1. Other (Specify) | | | | |  | | | | | | | | | | | | | |
|  | | For your last pregnancy, were you counseled about IFA supplementation? | | | | | 1. Yes 2. No | | | | | | | | | |  | | | |
|  | | For your last pregnancy, did you take IFA supplement? | | | | | 1. Yes 2. No | | | | | | | | | |  | | | |
|  | | Have you taken all doses IFA supplements as per the prescription? | | | | | 1. Yes 2. No | | | | | | | | | |  | | | |
|  | | For your last pregnancy, were you counseled about deworming medications? | | | | | 1. Yes 2. No | | | | | | | | | |  | | | |
|  | | For your last pregnancy, did you take deworming medications? | | | | | 1. Yes 2. No | | | | | | | | | |  | | | |
|  | | Are you immunized for Tetanus? | | | | | 1. Yes 2. No | | | | | | | | | |  | | | |
|  | | Where did you give birth to your last child? | | | | | 1. Home 2. Government Hospital 3. Government Health Center 4. Government Health Post 5. Private Hospital 6. Private Clinic 7. NGO Health facility 8. Others (Specify) | | | | | | | | | |  | | | |
|  | | Did you ever give live birth to LBW babies? | | | | | 1. Yes 2. No | | | | | | | | | |  | | | |
|  | | Number of babies born LBW  (both live or dead after live birth) | | | | | ________________________ | | | | | | | | | |  | | | |
|  | | Did you give birth to live Preterm babies? | | | | | 1. Yes 2. No | | | | | | | | | |  | | | |
|  | | Number of babies born Preterm  (both live or dead after live birth) | | | | |  | | | | | | | | | |  | | | |
|  | | Number of total live births | | | | |  | | | | | | | | | |  | | | |
|  | | Number of live children | | | | |  | | | | | | | | | |  | | | |
|  | | Have you attended postnatal care for your last delivery? | | | | | | 1. Yes 2. No | | | | | | | |  | | | | |
|  | | When did you attend postnatal care after your last delivery? | | | | | | 1. After 1 hour 2. Within 1 day 3. Within two days 4. After 2 days | | | | | | | |  | | | | |
|  | | During your last postnatal care, were you counseled about infant feeding methods? | | | | | | 1. Yes 2. No | | | | | | | |  | | | | |
|  | | Which feeding methods were you counseled about? | | | | | | 1-Yes | | | 2-No | | | | |  | | | | |
|  | | - 1. Breastfeeding | | | | | |  | | |  | | | | |  | | | | |
|  |  | - 1. Formula feeding | | | | | |  | | |  | | | | |  |  |  |  |  |
|  |  | - 1. Complementary feeding | | | | | |  | | |  | | | | |  |  |  |  |  |
|  |  | - 1. Bottle feeding | | | | | |  | | |  | | | | |  |  |  |  |  |
|  |  | - 1. Cow milk feeding | | | | | |  | | |  | | | | |  |  |  |  |  |
|  |  | - 1. Other (Specify) | | | | | |  | | | | | | | |  |  |  |  |  |
|  | | Are you currently doing something or using any method to delay or avoid getting pregnant? | | | | | | 1. Yes 2. No | | | | | | | |  | | | | |
| **Section V ; Mothers Knowledge, Attitude and Practice of Breast Feeding and complementary food** | | | | | | | | | | | | | | | | | | | | |
|  | | What are the common feeding practices for infants below 6 months in your community? (multiple answers possible) | | | | | | - - 1. Breastfeeding     2. Formula feeding     3. Wet nursing (feeding an infant the breast milk of another woman)     4. Cow milk feeding     5. Solid and semi-solid foods     6. Other liquid     7. Others (Specify) | | | | | | | |  | | | | |
|  | | What are the common feeding practices for children 6 months and above in your community?  (multiple answers possible) | | | | | | 1. Breastfeeding 2. Formula feeding 3. Wet nursing (feeding an infant the breast milk of another woman) 4. Cow milk feeding 5. Solid and semi-solid foods 6. Other liquid 7. Others (Specify) | | | | | | | |  | | | | |
|  | | Did you ever breastfeed your child? | | | | | |  | | | | | | | |  | | | | |
|  | | After delivery, when should the mothers start breast-feeding her infants? | | | | | | (Hours/Days/Weeks/Months) | | | | | | | |  | | | | |
|  | | When did your start breast-feeding your youngest children after delivery? | | | | | | (Hours/Days/Weeks/Months) | | | | | | | |  | | | | |
|  | | When did your start breast-feeding [the name of the child] after delivery? | | | | | | (Hours/Days/Weeks/Months) | | | | | | | |  | | | | |
|  | | During the first three days after delivery, did you give your children anything else to eat or drink? | | | | | | 1. Yes 2. No 3. Don’t know | | | | | | | |  | | | | |
|  | | During the first three days after delivery, did you give your [the name of the child] anything else to eat or drink? | | | | | | 1. Yes 2. No 3. Don’t know | | | | | | | |  | | | | |
|  | | For how long the mothers should only breastfeed her infants? | | | | | | (Months) | | | | | | | |  | | | | |
|  | | For how long did you only breastfeed your youngest child? | | | | | | (Months) | | | | | | | |  | | | | |
|  | | For how long did you only breastfeed [the name of the child]? | | | | | | (Months) | | | | | | | |  | | | | |
|  | | If you are currently breast-feeding, how many times in the last 24 hrs including day and night did you breastfeed? | | | | | |  | | | | | | | |  | | | | |
|  | | If you are currently breast-feeding, for how long are you planning to only breastfeed your child? | | | | | | (Months) | | | | | | | |  | | | | |
|  | | For how long the mothers should breastfeed her child with other foods? | | | | | | (Months) | | | | | | | |  | | | | |
|  | | For how long did you breastfeed your youngest child with other foods? | | | | | | (Months) | | | | | | | |  | | | | |
|  | | For how long did you breastfeed [the name of the child] with other foods? | | | | | | (Months) | | | | | | | |  | | | | |
|  | | If you are currently breast-feeding, for how long are you planning to breastfeed your child with other foods? | | | | | |  | | | | | | | |  | | | | |
|  | | What should the mothers feed her infants during the first 6 months? | | | | | | 1. Breast milk only 2. Breast milk with other fluids like cow milk 3. Breast milk with other fluids and foods 4. Others (Specify) | | | | | | | |  | | | | |
|  | | Do you believe that BF is important for infants? | | | | | | 1. Yes 2. No | | | | | | | |  | | | | |
|  | | What do you think is the importance of breast-feeding for the mother and the child? | | | | | | 1-Yes | | | | 2-No | | | |  | | | | |
|  |  | - 1. Mother-infant bonding | | | | | |  | | | |  | | | |  | | | | |
|  |  | - 1. Family planning | | | | | |  | | | |  | | | |  | | | | |
|  |  | - 1. Prevention of childhood disease | | | | | |  | | | |  | | | |  | | | | |
|  |  | - 1. Cheap | | | | | |  | | | |  | | | |  | | | | |
|  |  | - 1. Cleanliness | | | | | |  | | | |  | | | |  | | | | |
|  |  | - 1. Availability | | | | | |  | | | |  | | | |  | | | | |
|  |  | - 1. Others (Specify) | | | | | |  | | | | | | | |  | | | | |
|  | | Do you believe that breastfeeding alone is enough for the first six months for infant life? | | | | | | 1. Yes 2. No | | | | | | | |  | | | | |
|  | | When should the mother start feeding her child food other than breast feeding? | | | | | | (Months) | | | | | | | |  | | | | |
|  | | When did you start feeding food other than breast feeding for [the name of the child]? | | | | | |  | | | | | | | |  | | | | |
|  | | What should the mothers feed her infant after 6 months? | | | | | | 1. Breast milk only 2. Breast milk with other fluids like cow milk 3. Breast milk with other fluids and foods 4. Others (Specify) | | | | | | | |  | | | | |
|  | | Did you vaccinate your youngest child? | | | | | | 1. Yes 2. No | | | | | | | |  | | | | |
|  | | Did your youngest child completed vaccinations appropriate for his age? (check of there is vaccination card) | | | | | | 1. Yes 2. No | | | | | | | | Maternal report  Verified from card | | | | |
|  | | Did you vaccinate [the name of the child]? | | | | | | 1. Yes 2. No | | | | | | | |  | | | | |
|  | | Did [the name of the child] completed vaccinations appropriate for his age? (check of there is vaccination card) | | | | | | 1. Yes 2. No | | | | | | | | Maternal report  Verified from card | | | | |
|  | | Do you use a mosquito net at night for your child? | | | | | | 1. Yes 2. No | | | | | | | |  | | | | |
| **Section VI ; Child feeding practice and 24 hour dietary recall**  Ask about the frequency and consumption of the listed liquids, solid or semi-solid food that the child has taken yesterday during the day or night according to food group | | | | | | | | | | | | | | | | | | | | |
|  | Did he or she eat any solid, semi-solid or soft foods yesterday during the day or night? | | | | | 1. Yes 2. No | | | | | | | | | | | |  | | |
|  | How many times did he or she eat solid, semi-solid or soft foods yesterday during the day or night? | | | | |  | | | | | | | | | | | |  | | |
| **Code** | **Food groups** | | | | | **Food items** | | | | | | | | **Coding Categories** | | | | | | |
|  |  |  |  |  |  |  |  |  |  |  |  |  |  | 2 - Yes | | | | 1 - No | | |
|  | Does your child taken (Grains, Roots and Tubers) yesterday during the day or night? | | | | | Porridge, bread, rice and noodles | | | | | | | |  | | | |  | | |
|  | Does your child taken (Vitamin A rich fruits and vegetables) yesterday during the day or night? | | | | | Carrot and green leafy vegetables like cabbage | | | | | | | |  | | | |  | | |
|  | Does your child taken (Flesh foods) yesterday during the day or night? | | | | | Meat, beef, chicken and fish | | | | | | | |  | | | |  | | |
|  | Does your child taken (Other fruits and vegetables) yesterday during the day or night? | | | | | Banana, avocado, tomatoes, orange and apple | | | | | | | |  | | | |  | | |
|  | Does your child taken (Dairy product) yesterday during the day or night? | | | | | Yoghurt, cheese or other milk products | | | | | | | |  | | | |  | | |
|  | Does your child taken (Legumes and nuts) yesterday during the day or night? | | | | | Beans, peas, lentils, nuts or seeds | | | | | | | |  | | | |  | | |
|  | Does your child taken (Eggs) yesterday during the day or night? | | | | |  | | | | | | | |  | | | |  | | |
|  | Does your child ever drink anything from a bottle with a nipple? | | | | | 1. Yes 2. No 3. Don’t know | | | | | | | |  | | | |  | | |
|  | Does your child ever drink anything from a bottle with a nipple yesterday during the day or night? | | | | |  | | | | | | | |  | | | |  | | |
| **Section VII ; Household Food Insecurity Access Scale (HFIAS) Measurement Tool** | | | | | | | | | | | | | | | | | | | | |
|  | In the past four weeks, did you worry that your household would not have enough food? | | | | | 1. Yes 2. No | | | | | | | | | | | |  | | |
|  | How often did this happen? | | | | | 1. Rarely(once or twice in the past four weeks) 2. Sometimes(three to ten times in the past four weeks) 3. Often(more than ten times in the past four weeks) | | | | | | | | | | | |  | | |
|  | In the past four weeks, were you or any household member not able to eat the kinds of foods you preferred because of lack of resource? | | | | | 1. Yes 2. No | | | | | | | | | | | |  | | |
|  | How often did this happen? | | | | | 1. Rarely(once or twice in the past four weeks) 2. Sometimes(three to ten times in the past four weeks) 3. Often(more than ten times in the past four weeks) | | | | | | | | | | | |  | | |
|  | In the past four weeks, did you or any household member have to eat a limited variety of foods due to lack of resources? | | | | | 1. Yes 2. No | | | | | | | | | | | |  | | |
|  | How often did this happen? | | | | | 1. Rarely(once or twice in the past four weeks) 2. Sometimes(three to ten times in the past four weeks) 3. Often(more than ten times in the past four weeks) | | | | | | | | | | | |  | | |
|  | In the past four weeks, did you or any household member have to eat some foods that you really did not want to eat because of lack of resource to obtain other types of food? | | | | | 1. Yes 2. No | | | | | | | | | | | |  | | |
|  | How often did this happen? | | | | | 1. Rarely(once or twice in the past four weeks) 2. Sometimes(three to ten times in the past four weeks) 3. Often(more than ten times in the past four weeks) | | | | | | | | | | | |  | | |
|  | In the past four weeks, did you or any household member have to eat a smaller meal than you felt you needed because there was not enough food? | | | | | 1. Yes 2. No | | | | | | | | | | | |  | | |
|  | How often did this happen? | | | | | 1. Rarely(once or twice in the past four weeks) 2. Sometimes(three to ten times in the past four weeks) 3. Often(more than ten times in the past four weeks) | | | | | | | | | | | | |  | |
|  | In the past four weeks, did you or any other household member have to eat fewer meals in a day because there was not enough food? | | | | | 1. Yes 2. No | | | | | | | | | | | | |  | |

|  | How often did this happen? | 1. Rarely(once or twice in the past four weeks) 2. Sometimes(three to ten times in the past four weeks) 3. Often(more than ten times in the past four weeks) |  |
| --- | --- | --- | --- |
|  | In the past four weeks, was there ever no food to eat of any kind in your household because of lack of resources to get food? | 1. Yes 2. No |  |
|  | How often did this happen? | 1. Rarely(once or twice in the past four weeks) 2. Sometimes(three to ten times in the past four weeks) 3. Often(more than ten times in the past four weeks) |  |
|  | In the past four weeks, did you or any household member go to sleep at night hungry because there was not enough food? | 1. Yes 2. No |  |
|  | How often did this happen? | 1. Rarely(once or twice in the past four weeks) 2. Sometimes(three to ten times in the past four weeks) 3. Often(more than ten times in the past four weeks) |  |
|  | In the past four weeks, did you or any household member go a whole day and night without eating anything because there was not enough food? | 1. Yes 2. No |  |
|  | How often did this happen? | 1. Rarely(once or twice in the past four weeks) 2. Sometimes(three to ten times in the past four weeks) 3. Often(more than ten times in the past four weeks) |  |
