# Supplementary material for: The effects of psychosocial stimulation on the development, growth, and treatment outcome of children with severe acute malnutrition age 6–59 months in southern Ethiopia: a parallel group cluster randomized control trial (EPSoSAMC study)
Source: BMC Public Health. 2019 Dec 2;19:1610. doi: 10.1186/s12889-019-7916-5 (PMC6889618; doi:10.1186/s12889-019-7916-5)
Supplement: Supplementary file 2 — Additional file 2. Follow Up Data Collection Questionnaire [file 12889_2019_7916_MOESM2_ESM.docx]

Additional File 2: Follow Up Data Collection Questionnaire

| **Code** | **Questions and Filters** | | **Coding Categories** | | |  |
| --- | --- | --- | --- | --- | --- | --- |
| **General Information** | | | | | |  |
|  | Child's unique ID Number | |  | | |  |
|  | Name of the child | |  | | |  |
|  | Health Facility Name | |  | | |  |
|  | Name of the Home Visitor | |  | | |  |
|  | Name of Supervisor | |  | | |  |
|  | Date of the Home Visit | | (D/M/Y-LC) | | |  |
|  | Time of the Home Visit | | (Hr/min-LT) | | |  |
| **Basic Background Information of the Respondent** | | | | | |  |
|  | Name of respondent |  | | | |  |
|  | Is the respondent is the trained primary caregiver? | 1. Yes 2. No | | |  |  |
|  | Relationship with the child | 1. Mother 2. Father 3. Relatives (grandfather, grandmother, uncle, aunt ) 4. Foster mother/Family 5. Employed caregiver 6. Others (Specify) | | | |  |
| **Intervention/stimulation Related Information (only for Children Under the Intervention Group)** | | | | | |  |
|  | Visit round (Circle the Appropriate Number) | 1^st^……….2^nd^………3^rd^ ……..4^th^……..5^th^ | | | |  |
|  | Has the child been following the intervention continually? | 1. Yes 2. No | | | |  |
|  | Where the child has been engaged in play activities? | 1. At home ground 2. At neighbors home ground 3. On bed | | | |  |
|  | Mother/Primary caretaker –child interaction | 1. Yes 2. No | | | |  |
|  | Mother -child interaction time | 1. During feeding and toileting 2. Out of working hours 3. Always together | | | |  |
|  | Other family members-child interaction | 1. Yes 2. No | | | |  |
|  | Other family members-child interaction time | 1. During feeding and toileting 2. Out of working hours 3. Always together | | | |  |
|  | A person more often attached with the child | 1. Elder brother/ sister 2. Younger brother/sister 3. A child less than 11 years of age 4. Grandparents 5. The child is lonely 6. Other adult family member | | | |  |
|  | Do the child meet other children in neighbor? | 1. Yes 2. No | | | |  |
|  | Number of children the child meets in neighbor |  | | | |  |
|  | How often the child the child meets other children in neighbor? |  | | | |  |
|  | Time spent on play by the child | 1. Always 2. Sometimes 3. Very rarely 4. Does not play at all | | | |  |
|  | Facilities for play | 1. Has different play material 2. has play corner arranged for him/ her 3. Pays cultural dances, songs and riddles with family | | | |  |
|  | How often does the trained primary caregiver engage the child in play/stimulation? | 1. Always 2. Sometimes 3. Never interacts with others | | | |  |
|  | How often has the child been getting guided play each day? | 1. Once 2. Twice 3. More than twice | | | |  |
|  | Do family members other than the trained caregivers are taking part in stimulation of the child? | 1. Yes 2. No | | | |  |
|  | Whom are the other family members taking part in stimulation of the child? | 1. Mother 2. Father 3. Other Children 4. Other adults in the Family 5. Employed caregiver 6. Others (Specify) | | | | |
|  | How often other family members are taking part in stimulation of the child? | 1. Always 2. Sometimes 3. Rarely 4. Not at all | | | | |
|  | Has the child been interested in attending the play sessions? | 1. Yes 2. No | | | | |
|  | Was the child sick after discharge from the health facility or after the previous home visit? | 1. Yes 2. No | | | | |
|  | If yes, the type of illness |  | | | | |
|  | If yes, was the child taken to the nearby health center for the sickness? | 1. Yes 2. No | | | | |
| 1. I | Is he/she given any medication |  | | | | |
|  | Did the child stop the stimulation/play because of sickness? | 1. Yes 2. No | | | | |
|  | If yes, for how many days did the child stop the stimulation/play? |  | | | | |
|  | After discharge from the SC /last home visit, was the child taken to the nearby health center for follow up? | 1. Yes 2. No | | | | |
|  | What are you feeding the child? | 1. Especially prepared home food 2. Whatever is available for the family at home 3. Cow milk 4. Powdered milk | | | | |
|  | Has the child been getting plumpy nut (BP 100) from nearby health center after discharge / last home visit? | 1. Always 2. Sometimes 3. Rarely 4. Not at all | | | | |
|  | What are the major challenges faced so far in working on the child? | | | | | |
|  | What do you think should be done to make the child support more effective/interesting? | | | | | |
|  | What did you like most so far while working with the child? | | | | | |
|  | What is your overall comments/suggestions on what is being done to your child? | | | | | |
| **Health Education** | | | | | | |
|  | The topic of Health Education for the visit | | |  | | |
|  | Time taken for the Health Education | | |  | | |
|  | Do family members other than the respondent are participated in Health Education session? | | |  | | |
|  | Number of family members other than the respondent participated in Health Education? | | |  | | |

| **Observation** | |
| --- | --- |
|  | The home visitors rating of the nature of the home environment for the child stimulation   1. Excellent – the home environment is appropriate for the child stimulation where play materials, and adult guidance are available and the child safety issues are well ensured 2. Acceptable– the home environment is somewhat appropriate for the child stimulation where some play materials, and some adult guidance are available and the child safety issues are somewhat ensured 3. Poor - the home environment is not appropriate for the child stimulation where no play materials, and adult guidance are available and the child safety issues are not ensured |
